# Subclinical epileptiform activity in the Alzheimer continuum: association with disease, cognition and detection method

**DOI:** 10.1186/s13195-023-01373-9

**Published:** 2024-01-23

**Authors:** Amber Nous, Laura Seynaeve, Odile Feys, Vincent Wens, Xavier De Tiège, Pieter Van Mierlo, Amir G. Baroumand, Koenraad Nieboer, Gert-Jan Allemeersch, Shana Mangelschots, Veronique Michiels, Julie van der Zee, Christine Van Broeckhoven, Annemie Ribbens, Ruben Houbrechts, Sara De Witte, Mandy Melissa Jane Wittens, Maria Bjerke, Caroline Vanlersberghe, Sarah Ceyssens, Guy Nagels, Ilse Smolders, Sebastiaan Engelborghs

**Affiliations:** 1 Department of Neurology, Universitair Ziekenhuis Brussel (UZ Brussel), Brussels, Belgium; 2 Neuroprotection and Neuromodulation (NEUR) Research Group, Center for Neurosciences, Vrije Universiteit Brussel, Laarbeeklaan 103, Brussels, Belgium; 3 Department of Biomedical Sciences, Universiteit Antwerpen, Antwerp, Belgium; 4 Laboratory of Pharmaceutical Chemistry, Drug Analysis and Drug Information (FASC), Research Group Experimental Pharmacology (EFAR), Center for Neurosciences, Vrije Universiteit Brussel (VUB), Brussels, Belgium; 5 Department of Neurology, Université Libre de Bruxelles (ULB), Hôpital Universitaire de Bruxelles (HUB), Hôpital Erasme, Brussels, Belgium; 6 Laboratoire de Neuroimagerie Et Neuroanatomie Translationnelles (LN2T), Université Libre de Bruxelles (ULB), ULB Neuroscience Institute (UNI), Brussels, Belgium; 7 Department of Translational Neuroimaging, Université Libre de Bruxelles (ULB), Hôpital Universitaire de Bruxelles (HUB), Hôpital Erasme, Brussels, Belgium; 8 Epilog NV, Ghent, Belgium; 9 Department of Radiology, Universitair Ziekenhuis Brussel, Vrije Universiteit Brussel, Brussels, Belgium; 10 Neurodegenerative Brain Diseases, VIB Center for Molecular Neurology, Antwerp, Belgium; 11 Icometrix, Louvain, Belgium; 12 Department of Clinical Biology, Laboratory of Clinical Neurochemistry, Universitair Ziekenhuis Brussel, Brussels, Belgium; 13 Department of Anaesthesiology and Perioperative Medicine, Universitair Ziekenhuis Brussel (UZ Brussel), Brussels, Belgium; 14 Department of Nuclear Medicine, Universitair Ziekenhuis Antwerpen, University of Antwerp, Antwerpen, Belgium; 15 Artificial Intelligence Supported Modelling in Clinical Sciences (AIMS) Research Group, Center for Neurosciences, Vrije Universiteit Brussel, Brussels, Belgium

**Keywords:** Alzheimer’s disease, Subclinical epileptiform activity, Interictal epileptic discharges, Magnetoencephalography, Long-term electroencephalography, High-density electroencephalography

## Abstract

**Background:**

Epileptic seizures are an established comorbidity of Alzheimer’s disease (AD). Subclinical epileptiform activity (SEA) as detected by 24-h electroencephalography (EEG) or magneto-encephalography (MEG) has been reported in temporal regions of clinically diagnosed AD patients. Although epileptic activity in AD probably arises in the mesial temporal lobe, electrical activity within this region might not propagate to EEG scalp electrodes and could remain undetected by standard EEG. However, SEA might lead to faster cognitive decline in AD.

**Aims:**

1. To estimate the prevalence of SEA and interictal epileptic discharges (IEDs) in a well-defined cohort of participants belonging to the AD continuum, including preclinical AD subjects, as compared with cognitively healthy controls.

2. To evaluate whether long-term-EEG (LTM-EEG), high-density-EEG (hd-EEG) or MEG is superior to detect SEA in AD.

3. To characterise AD patients with SEA based on clinical, neuropsychological and neuroimaging parameters.

**Methods:**

Subjects (*n* = 49) belonging to the AD continuum were diagnosed according to the 2011 NIA-AA research criteria, with a high likelihood of underlying AD pathophysiology. Healthy volunteers (*n* = 24) scored normal on neuropsychological testing and were amyloid negative. None of the participants experienced a seizure before. Subjects underwent LTM-EEG and/or 50-min MEG and/or 50-min hd-EEG to detect IEDs.

**Results:**

We found an increased prevalence of SEA in AD subjects (31%) as compared to controls (8%) (*p* = 0.041; Fisher’s exact test), with increasing prevalence over the disease course (50% in dementia, 27% in MCI and 25% in preclinical AD). Although MEG (25%) did not withhold a higher prevalence of SEA in AD as compared to LTM-EEG (19%) and hd-EEG (19%), MEG was significantly superior to detect spikes per 50 min (*p* = 0.002; Kruskall–Wallis test). AD patients with SEA scored worse on the RBANS visuospatial and attention subset (*p* = 0.009 and *p* = 0.05, respectively; Mann–Whitney *U* test) and had higher left frontal, (left) temporal and (left and right) entorhinal cortex volumes than those without.

**Conclusion:**

We confirmed that SEA is increased in the AD continuum as compared to controls, with increasing prevalence with AD disease stage. In AD patients, SEA is associated with more severe visuospatial and attention deficits and with increased left frontal, (left) temporal and entorhinal cortex volumes.

**Trial registration:**

Clinicaltrials.gov, NCT04131491. 12/02/2020.

## Background

Epileptic seizures have been described as a clinical feature that could be present in patients with advanced stages of probable Alzheimer’s disease (AD), according to the National Institute of Neurological and Communicative Diseases and Stroke/Alzheimer's Disease and Related Disorders Association (NINCDS-ADRDA) criteria of 1984 [1]. Ongoing research confirmed epileptic seizures to be a comorbidity of AD, with 10–22% of AD patients having at least one epileptic seizure during the course of their disease [2]. Current literature furthermore suggests that seizures can occur early in the time course of the disease, possibly before or concurrent with symptom onset [3, 4]. Amyloid-beta (Aβ) and tau, hallmark AD proteins that are present years before the appearance of clinical AD symptoms [5], might play a role in the development of neuronal hyperactivity [6]. Preclinical work showed that a fraction of cortical and hippocampal neurons become hyperactive in the vicinity of Aβ plaque-enriched regions in old AD mice [7, 8]. Furthermore, a selective increase in hyperactive neurons in the hippocampus of young mice has been found before the formation of plaques, suggesting a role for soluble Aβ [8]. Reducing endogenous tau in non-transgenic mice and transgenic amyloid precursor protein (APP) mice has been shown to decrease spontaneous seizures and the severity of chemically induced seizures [9]. Vande Vyver et al. found increased susceptibility for seizures and kindling in mice with mutations increasing Aβ only or both Aβ and tau in the brain [10].

Next to an increased seizure risk, an increased prevalence of subclinical epileptiform abnormalities has been described in clinically diagnosed AD patients [11–13]. Lam et al. found a prevalence of epileptiform abnormalities as measured by 24-h EEG recordings in 53% of AD patients with epilepsy (AD-Ep) and 22% of AD patients with no history or risk factors for epilepsy (AD-NoEp), whereas this prevalence was only 4.7% in healthy controls [11]. Vossel et al. found an increased prevalence of subclinical epileptiform activity (SEA) in AD patients (42%) as compared to healthy volunteers (10.5%) by use of magnetoencephalography (MEG) and long term-EEG (LTM-EEG) monitoring [12]. Horvath et al. found subclinical epileptiform discharges in 54% of AD patients versus in 25% of healthy controls by use of LTM-EEG recordings [13]. Since neuronal hyperactivity and epileptic seizures might be present even before or concurrent with AD symptom onset [3, 4, 8], the first aim of our study was to evaluate the prevalence of SEA in the AD continuum, even including the preclinical AD stage, as compared to healthy controls. Therefore, we used well-defined cohorts, with biomarker-based diagnoses, reflecting a high likelihood of underlying AD pathophysiology [5, 14, 15].

Seizure risk is higher in patients with autosomal dominant AD: a seizure frequency of 47.7%, after mean follow up of 8.4 years was found in AD patients harbouring a pathogenic *PSEN1*, *PSEN2*, *APP* mutation or a duplication of *APP* [16]. A recent meta-analysis showed that the ε4 allele of *APOE*, a known risk factor for AD, is also a risk factor for epilepsy with the epilepsy risk increasing with the number of ε4 copies [17]. Younger AD patients were found to be more likely to have unprovoked seizures, with a 87-fold increase in the age group of 50–59 years as compared to the age-matched general population [18]. Epilepsy has been stated as a frequent comorbidity in early-onset AD [19]. A recent study by Horvath et al. confirmed that AD patients with epileptic discharges on EEG and/or epileptic seizures had a dementia onset at a younger age. They also had more years of education and performed worse on the Addenbrooke Cognitive Examination (ACE) score or had higher Verbal Fluency + Language scores ratio, as compared to AD patients without epileptic seizures or discharges. AD patients with epileptic seizures had a longer duration of dementia, as compared to patients without epileptic seizures or with epileptic discharges [20]. Another study by the same group showed that AD patients with seizures had worse visuo-spatial scores and had smaller parietal thickness associated with reduced thickness of the precunei as compared to those without seizures [21]. Hahm et al. found smaller volumes in the right parahippocampal gyrus, left angular gyrus and middle temporal gyrus in a group of AD patients with seizures as compared to the AD group without seizures [22]. Since SEA might lead to disease progression [12, 13, 23–25] and as such might serve as potential treatment strategy, it could become of importance to identify AD patients with SEA. Therefore, we wanted to compare clinical, neuropsychological and neuroimaging measures between AD patients with SEA and those without, in order to set up future studies that would allow us to predict SEA based on these data.

SEA in AD was found to mostly arise in the temporal lobes in previously mentioned studies [11–13]*.* Based on seizure semiology, it is known that seizures in AD mostly arise from the mesial temporal lobe [26]. This is one of the first structures affected by AD pathology and the most epileptogenic region in the brain [4, 26]. Epileptiform activity can be recorded using two modalities: EEG and MEG. While both EEG and MEG measure signals generated by neuronal currents, they each have their own (in)sensitivities and offer complementary insights. Whereas the layered structure of the head tissues leads to different electrical conductivities that smear electrical signals, magnetic fields are unaffected and pass without much distortions. For this reason, MEG has a higher spatial resolution than EEG, at least for superficially arising signals. On the other hand, EEG is sensitive to radial and tangential currents while MEG is mostly sensitive to tangential currents, and EEG offers higher sensitivity to (strong) deep currents [27–29]. The current 10–20 EEG system furthermore insufficiently records activity arising from anterior, mesial and inferior-basal parts of the temporal lobe, which make up the mesial temporal structures. This is why the *International Federation of Clinical Neurophysiology (IFCN)* proposes to add the inferior temporal chain from the 10–10 system, including T9/T10, F9/F10 and P9/P10 to the standard EEG recording [30]. It has been shown that in 59% of patients with temporal lobe abnormalities on EEG, the peak electronegativity was seen over the inferior temporal chain, and in 6% of them, the electronegativity was only seen over this inferior temporal chain [31]. In our current study, we wanted to evaluate whether one neurophysiological technique (LTM-EEG versus high-density EEG [hd-EEG] with inferior temporal chain versus MEG) would be superior to detect SEA in AD, the more so as hd-EEG has not been used in AD before.

The aims of our study were to compare the detectability of SEA and the number of interictal epileptic discharges (IEDs) in a well-defined cohort of participants belonging to the AD continuum (including preclinical AD subjects) with cognitively healthy controls, to evaluate whether LTM-EEG, hd-EEG or MEG is superior to detect SEA in AD and to characterise AD patients with SEA based on clinical, neuropsychological and neuroimaging parameters.

## Methods

### Subjects

Eight patients with dementia due to AD, 33 patients with MCI due to AD, 8 subjects with preclinical AD and 24 cognitively healthy volunteers were included in a prospective, observational study at the Department of Neurology of the University Hospital Brussels (UZ Brussel) and at the Department of Urology and Anaesthesia of the University Hospital Brussel (UZ Brussel). The study was approved by the local Ethics Committee (UZ Brussel – BUN 143201941207). All participants, or their legal representatives in case of dementia, gave written informed consent. Participants were included in the study between February 2020 and May 2023.

#### Diagnostic criteria

##### AD patients (MCI and dementia)

We selected patients with a diagnosis of MCI and probable AD based on the 2011 National Institute on Ageing and Alzheimer’s disease (NIA-AA) criteria. Patients met the 2011 research criteria using biomarker-based diagnoses, reflecting high probability of underlying AD pathophysiology. In summary, all subjects had a positive biomarker reflecting Aβ deposition in the brain (positron emission tomography [PET] amyloid positivity or low cerebrospinal fluid [CSF] Aβ_1-42_/Aβ_1-40_ ratio) and a positive biomarker reflecting neuronal injury (increase in CSF T-tau [*n* = 35 out of 38 AD patients who had lumbar puncture (LP)] or P-tau [*n* = 36 out of 38 AD patients who had LP] and/or hippocampal atrophy on MRI and/or temporoparietal hypometabolism on fluorodeoxyglucose [FDG]-PET) [14, 32]. Amongst those who had LP, there was only one patient who did not have a T-tau nor P-tau increase in CSF. This patient did have hippocampal atrophy on MRI and temporoparietal hypometabolism on FDG-PET. Amongst the three AD patients that had amyloid PET scan (instead of LP for CSF biomarker analysis), one had temporoparietal hypometabolism on FDG-PET and normal MRI, one had hippocampal atrophy on MRI and normal FDG-PET and one had both temporoparietal hypometabolism on FDG-PET and hippocampal atrophy on MRI. Whereas the 2011 NIA-AA criteria describe low CSF Aβ_1-42_ levels as a positive Aβ biomarker, we used low CSF Aβ_1-42_/Aβ_1-40_ ratio in order to increase diagnostic accuracy [33].

##### Preclinical AD subjects

Subjects with preclinical AD met the 2011 NIA-AA criteria. They had normal scores on their neuropsychological testing but showed evidence of a positive Aβ biomarker (brain PET amyloid positivity or low CSF Aβ_1-42_/Aβ_1-40_ ratio) with or without evidence of a biomarker for neuronal injury (increase in CSF T-tau or P-tau and/or hippocampal atrophy on MRI and/or temporoparietal hypometabolism on brain FDG-PET scan) [5]. Consistent with the approach applied to the AD patients, low CSF Aβ_1-42_/Aβ_1-40_ ratio levels were used in order to increase diagnostic accuracy [33].

##### AD group vs AD patients

The *AD group* represents all participants belonging to the AD continuum. The AD group thus consists of all patients with dementia due to AD, all patients with MCI due to AD and all preclinical AD subjects. The AD patients, on the other hand, only include patients with dementia due to AD and patients with MCI due to AD, but not the preclinical AD subjects.

##### Cognitively healthy controls

Healthy controls had normal scores on neuropsychological testing, as well as a normal CSF Aβ_1-42_/Aβ_1-40_ ratio or a negative PET-amyloid scan.

Healthy controls were recruited through the memory consultation at UZ Brussel (family members of AD patients; participants presenting with subjective cognitive decline) and through the Urology Department of UZ Brussel (patients who needed to undergo surgery under lumbar anaesthesia for urological pathologies).

#### Exclusion criteria

Participants could not have been diagnosed with epilepsy or seizures, have suffered a stroke (ischemic or haemorrhagic) or tissue-positive transient ischemic attack (TIA), suffer from alcohol or other substance abuse, have severe systemic medical illnesses, have normal pressure hydrocephalus or Korsakoff syndrome, and have other neurodegenerative diseases than AD with influence on cognition. Participants could not be younger than 45 years of age. They could not be under treatment with antiseizure medications. Benzodiazepine treatment for other indications than epilepsy/seizures was allowed.

### Study procedures

#### Neuropsychological testing

A battery of neuropsychological tests was performed in all participants by experienced neuropsychologists (S.M. and V.M.). These tests were performed in the participant’s mother tongue (French or Dutch). Neuropsychological examination consisted of following tests: Repeatable Battery for the Assessment of Neuropsychological Status (RBANS) [range: 40–160, lower scores indicate worse cognition [34], Geriatric Depression Scale (GDeprS) [range: 0–30, higher scores indicate more depression [35], Addenbrooke’s Cognitive Examination-Revised (ACE-R) [range: 0–100, lower scores indicate worse cognition [36], MMSE (as part of ACE-R) [range: 0–30, lower scores indicate worse cognition [37], Trail Making Test Part A and Part B (TMT-A and TMT-B) [times, higher times indicate worse cognition [38] and activities of daily living (ADL). Additionally, the Pittsburgh Sleep Quality Index (PSQI) [range: 0–21, higher scores indicate worse sleep [39] was completed by A.N. at the start of every LTM-EEG.

#### Biomarker analysis

##### CSF analysis of AD biomarkers

CSF collection via LP was performed in 46 participants (8 dementia, 30 MCI, 3 preclinical AD, 5 healthy controls) before or at inclusion in the study, for the purpose of AD biomarker analysis. Biomarkers were analysed using an automated enzyme-linked immunosorbent assay (ELISA) or chemiluminescence immunoassay (CLIA) (EUROIMMUN Analyzer I-2P or Lumipulse G600II/Fujirebio) at the UZ Brussel Lab of Neurochemistry. In some participants (*n* = 5), LP was performed before inclusion in the current study in the context of clinical routine practice; in these cases, CSF biomarker analysis was performed at the UAntwerp BIODEM laboratory using either an automated EUROIMMUN ELISA or manual ELISA (INNOTEST® -Amyloid1-42, INNOTEST® hTau-Ag, and INNOTEST® Phospho-Tau181P, respectively; Fujirebio Europe, Ghent, Belgium). Analysed CSF biomarkers include P-tau181, T-tau, Aβ1-42 and Aβ1-40. Values were all interpreted according to in-house validated cut-off values and intra- and interassay coefficients of variability were below 10%.

Patients (dementia, MCI) had their LP performed maximally 3 years and 4 months before neuropsychological testing with one exception, who underwent LP already 6 years before neuropsychological testing. Cognitively intact subjects (healthy controls, preclinical AD subjects) had their LP performed maximally 1 year and 3 months before neuropsychological testing.

##### Amyloid PET scan

A [^11^C]Pittsburgh compound-B (PIB) PET scan was performed at the Nuclear Medicine Department of Antwerp University Hospital (UZA) in 27 participants (3 MCI, 5 preclinical AD, 19 healthy controls) at inclusion in the current study. The [^11^C]PIB was acquired on a GE Discovery MI PET/CT scanner (3 or 4 ring scanner). All PET images were corrected for random and scattered coincidences and attenuated based on a delayed coincidence window and a low dose computed tomography (CT), respectively.

Patients (dementia, MCI) had their [^11^C]PIB scan performed maximally 7 months before neuropsychological testing, cognitively intact subjects (healthy controls, preclinical AD subjects) had their [C11]-PIB scan performed maximally 10 months after neuropsychological testing.

#### Genetic profiling and APOE genotyping

*APOE* genotyping and mutation screening was performed at the VIB-UAntwerp Center for Molecular Neurology after collection of 40 ml blood samples in each participant. The samples of participants were tested for several Mendelian genes associated with neurodegenerative diseases (*APP, PSEN1, PSEN2, MAPT, GRN, ABCA7* and *APOE genotype*) using an exome-based gene panel. Whole exome sequencing (WES) was performed using KAPA Hyper Prep and SeqCap WES solution according to the manufacturer’s protocols (Roche). Capture was performed on four pooled libraries, hybridising with exome library probes for 48 h. Three captures, all with different indexes, were equimolarly pooled and sequenced using NextSeq500 using NextSeq HO 300 sequencing chemistry (Illumina). Likely pathogenic variants were validated by Sanger sequencing. *APP* duplication was evaluated by use of multiplex amplicon quantification (MAQ) as previously described [40].

#### Brain MRI

##### MRI

Structural imaging was acquired in 67 participants, either on a research-dedicated 3 T hybrid PET-MR scanner (SIGNA (TM), GE Healthcare, Chicago, IL) at the CUB Hôpital Erasme (Brussels, Belgium) using whole-brain axial 3D T1 sequence, or on a Discovery MR750w 3 T (GE Medical Systems, Milwaukee, WI, U.S.A.) or 3 T Ingenia (Philips Medical Systems, Best, Netherlands) at Universitair Ziekenhuis Brussel (UZ Brussel) using sagittal 3D T1-weighted (T1w) MR sequence and a sagittal 3D fluid attenuated inversion recovery (FLAIR) sequence. Some patients had already undergone a brain MRI for clinical routine use or for another research protocol. In that case, the scanner used was the 1.5 T Achieva dStream (Philips Medical Systems, Best, The Netherlands) or 3 T Skyra (Siemens Medical Solutions, Pennsylvania, U.S.A.) with sagittal 3D T1-weighted (T1w) MR sequence and a sagittal 3D fluid attenuated inversion recovery (FLAIR) sequence. For one participant, MRI was performed in another hospital using a sagittal 3D T1-weighted (T1w) MR sequence. All MRI scans were performed within 1 year after neuropsychological testing, except for one MCI patient who had the MRI 14 months after neuropsychological testing.

##### Volumetric analysis

Subsequently, the acquired MRI scans were used for projection of MEG data into source space. All MRI scans were furthermore processed with icobrain dm (version 5.0; Icometrix, Leuven, Belgium) for an automated volumetric analysis of global and local brain region volumes [41, 42]. In short, after skull stripping and bias field correction, the icobrain pipeline performs an initial segmentation into grey matter, white matter and CSF (including white matter hyperintensities (WMH) if a FLAIR is available). This step is further refined to obtain sub-segmentations such as the hippocampi and cortical grey matter volumes. For our analysis, we used all volumes normalised for head size. The volumes analysed were the following: T1-hypointensities, FLAIR hyperintensities, whole brain, grey matter, cortical grey matter, white matter, frontal, parietal, temporal and occipital cortices, cingulate, anterior cingulate, and posterior cingulate cortices, entorhinal cortex, as well as hippocampal, parahippocampal, precuneus and fusiform volumes. If applicable, left and right-side volumes were calculated separately. FLAIR hyperintensities were furthermore analysed specifically in the posterior and posterior periventricular regions. The posterior region is defined as the dorsal part of the brain when a coronal section in the middle of the corpus callosum is made.

#### EEG and MEG

##### LTM-EEG

Seventy-one participants (8 dementia, 32 MCI, 8 preclinical AD and 23 healthy controls) underwent LTM-EEG monitoring with a median EEG time of 23.5 h and with a median artefact-free time of 18 h. The amount of artefact in each LTM-EEG was analysed using EEGLAB's *Automatic Continuous Rejection* function. Part of the participants (*n* = 44) underwent this EEG monitoring at home, using a 24-channel mobile system (mBrainTrain LLC, Belgrade, Serbia; http://www.mbraintrain.com/) attached to an elastic electrode cap (EASYCAP GmbH, Inning, Germany; http://www.easycap.de). Twenty-four Ag/AgCl electrodes were positioned at standard 10–20 locations. Nine participants had a 24-h EEG with electrodes placed at EOG1, EOG2, F7, F3, Fz, F4, F8, T7, C3, Cz, C4, T8, P7, P3, Pz, P4, P8, O1, O2, M1, M2, ECG1, ECG2 and ECG3. However, after internal revision putting emphasis on the importance of looking at the frontal regions as well, new caps were ordered with electrodes placed at Fp1, Fp2, F7, F3, Fz, F4, F8, T7, C3, Cz, C4, T8, Cpz, P7, P3, Pz, P4, P8, O1, O2, M1, M2, ECG1, and ECG2, which were used in the other 35 participants. Reference and ground electrodes were placed at FCz and AFz sites. The wireless EEG DC amplifier (weight = 60 g; size = 82 × 51 × 12 mm; resolution = 24 bit; sampling rate = 250 Hz, 0–250 Hz pass-band) was placed on the shoulder of the participant and sent digitised EEG data via Bluetooth to a Samsung smartphone, placed in the vicinity of the participant. Another participant subgroup (*n* = 27) underwent 24-h EEG monitoring at the sleep clinic of Universitair Ziekenhuis Brussel (UZ Brussel), using the Brainnet 3 device from Medatec (Haillot, Belgium, sampling rate = 200 Hz, 0.16 Hz–70 Hz pass-band). Disposable AgCl cup electrodes were used and positioned at standard 10–20 locations, being Fp1, Fp2, F7, F3, Fz, F4, F8, T7, C3, Cz, C4, T8, Cpz, P7, P3, Pz, P4, P8, O1, O2, M1, M2, ECG1, and ECG2. In this setting, participants were connected to the amplifier using a connection cable. Time between neuropsychological testing and LTM-EEG was maximally 9 months.

##### hd-EEG

Thirty-nine participants (2 dementia, 19 MCI, 5 preclinical AD and 13 healthy controls) underwent 50 min of resting-state hd-EEG-monitoring in a sleep-promoting environment (darkened room, laying down, encouraged to fall asleep) (Geodesic EEG System 400 with 128-channels MicroCel sensor nets, EGI Electrical Geodesics, Eugene, USA; low-pass: 450 ​Hz; sampling frequency: 1 ​kHz) at the CUB Hôpital Erasme (Brussels, Belgium), some of them simultaneously with MEG (*n* = 10). The 128 electrodes were based on low profile, AgCl-plated carbon-fibre electrode pellets specifically designed to avoid EEG-induced magnetic artefacts. Further, electrodes were only 2-mm thick so they minimally hampered head positioning in the MEG helmet. The reference electrode was placed at Cz and all impedances were kept below 50 ​kΩ thanks to a conductive gel between each electrode and the skin. Time between neuropsychological testing and hd-EEG was maximally 16 months.

##### Magnetoencephalography (MEG)

Twenty-three participants (3 dementia, 10 MCI, 3 preclinical AD, 7 healthy controls) underwent 50 min of resting-state MEG in a sleep-promoting environment (darkened room, laying down, encouraged to fall asleep) at the CUB Hôpital Erasme (Brussels, Belgium). Neuromagnetic activity was recorded (band-pass: 0.1–330 ​Hz, sampling frequency: 1 ​kHz) with a 306-channel whole-scalp MEG system installed in a lightweight magnetically shielded room (Maxshield™, Elekta Oy, Helsinki, Finland; now MEGIN). Subjects were scanned with a Neuromag Triux™ MEG (MEGIN, Cronton Healthcare, Helsinki, Finland), some of them simultaneously with hd-EEG (*n* = 10). Four coils tracked subjects’ head position inside the MEG helmet. The location of the coils with respect to anatomical fiducials were determined with an electromagnetic tracker (Fastrak, Polhemus, Colchester, VT, USA) prior to MEG recording. Time between neuropsychological testing and MEG was maximally 12 months.

##### EEG readout (LTM-EEG and hd-EEG)

LTM-EEG files and hd-EEG files were shared with Epilog (Ghent, Belgium) for automated spike detection. A spike was defined as a pointed transient, clearly distinguishable from EEG background, and usually having negative polarity relative to other scalp areas, with a duration of 20–70 ms [43]. Spikes were automatically detected using Persyst Spike Detector P14 (Persyst, San Diego, CA, USA). The spikes were detected during the whole EEG, including spike bursts and ictal events, both clinical and subclinical. The EEGs were then reviewed with the following settings: page speed was set at 30 mm/s, band-pass filter between 0.1 and 70 Hz with 50 Hz notch filter. Since automated spike detection is highly sensitive but less specific [44], all spikes annotated by the algorithm were evaluated by A.N. to eliminate artefacts (eye, heart, muscle, electrode), and spikes not clearly differentiated from background rhythms, using both the Average montage and Bipolar “double banana” montage for LTM-EEG, and Bipolar “triple banana” montage and its corresponding Average montage for hd-EEG. Ten-second epochs around each spike were then examined by a clinical epileptologist (L.S.) blind to diagnosis, using the same montages. Spikes were scored as being epileptic or not based upon this expert opinion. Spikes annotated as epileptiform discharges did not have characteristics of normal variants (e.g. positive occipital sharp transients of sleep, wicket spikes, V-waves…). Localisation of IED was based on the EEG electrode(s) over which phase reversal was seen in Bipolar montage and on which the highest amplitude was seen in Average montage, upon visual analysis.

##### MEG readout

Continuous MEG data were pre-processed off-line using a signal space separation (SSS) method, and if necessary its spatiotemporal extension (tSSS) (MaxFilter 2.2; with default parameters; MEGIN) to supress residual interference and correct for head movements [45]. High-pass filter was set at 3 Hz and low-pass filter at 40 Hz (as commonly used for IEDs detection [46]). The readout was performed according to recommendations of the IFCN by detection of spiky events popping out of the ongoing cerebral activity [47]. IEDs were then manually identified by visual data inspection (in agreement with current clinical practice in Europe [48]) based on usual IED waveforms [49] and duration (i.e. between 20 and 120 ms [50]) and clear dipolar magnetic field patterns [51] by two trained observers (O.F., resident in neurology trained to MEG readout, and X.D.T., clinical magnetoencephalographer with 15 years of experience). Dipole modelling was performed to assess the spatial distribution of the MEG data, the physiological pattern of the dipole, its cortical/juxtacortical location and its goodness-of-fit [47] with a cut-off fixed at 80% [52]. Additionally, source reconstruction (equivalent current dipole modeling based on spherical head model) was performed at the peak of each individual spiky events to localise IEDs [29] and to improve the distinction between IEDs and normal variants (e.g. sharp perisylvian magnetoencephalography transients [53]). Normal variants as well as physiological activity were therefore rejected. IEDs were confirmed after consensus agreement between O.F. and X.D.T. who were blinded to diagnosis and counted by A.N.

##### Subclinical epileptiform activity (SEA)

Since we do not have a MEG, hd-EEG and LTM-EEG investigation in each participant, we considered that a participant exhibits SEA if IEDs were identified on either MEG and/or hd-EEG and/or LTM-EEG.

### Analysis

#### Formulas

The following formula is only used for Table 3 to compare spikes between MEG, hd-EEG and LTM-EEG. During LTM-EEG, certain parts are susceptible to the presence of artefacts, varying between different participants. To compare spike counts between our different techniques (LTM-EEG vs hd-EEG vs MEG), we controlled for artefacts on LTM-EEG. Furthermore, we compared spikes between our different techniques as calculated per 50 min (as this is the exact timing of hd-EEG and MEG). For LTM-EEG, after artefact rejection, we divided the number of spikes in the recording by the number of minutes in the recording and then multiplied that fraction by 50 to get the average number of spikes per period of 50 min, making the value comparable to the EEG and MEG measurements that lasted 50 min.

#### Statistical analyses

Analyses were performed using the SPSS®29.0 software package. Values are reported as either exact number or median with interquartile range (IQR), displayed as [Q1;Q3].

Due to the limited sample size, nonparametric tests were applied. To detect differences in categorical binary data between groups, the Fisher’s exact test was used (gender, cardiovascular risk factors, medication intake, *APOE* status, prevalence of SEA). Mann–Whitney *U* tests were applied for pairwise comparisons and Kruskall–Wallis tests for comparison between multiple groups (with Mann–Whitney *U* tests and Bonferroni correction for post-hoc) in continuous data (age, neuropsychological testing scores, brain volumes, biomarker values). The significance level was set to 0.05. Post-hoc Bonferroni correction was used when comparing between multiple groups. Since we compared multiple variables between two groups (AD patients with SEA versus those without), we did not apply Bonferroni correction for multiple testing as this would lead to very stringent *p*-values.

TMT part A and B are reported in times, when participants could not perform the TMT part B within 300 s, their time was equated to 300 s.

GraphPad Prism®9 software package was used for graphical representations.

## Results

### Baseline characteristics

An overview of baseline participant characteristics is given in Table 1*.* There were no significant differences regarding sex, age, years of education or handedness between our groups. MMSE and ACE-R scores were significantly lower in AD patients than both healthy controls and preclinical AD subjects. AD patients had lower whole brain volumes than healthy controls and lower hippocampal volumes than both preclinical AD subjects and healthy controls. On the other hand, AD patients had higher FLAIR hyperintensity volumes as compared to healthy controls. Absence of *APOE* ε4 alleles was more frequent in healthy controls than AD patients and preclinical AD subjects. Preclinical AD subjects had significantly higher prevalence of hypertension than healthy controls. There were no significant differences regarding other cardiovascular risk factors between our groups, nor were there differences with regarding GDeprS or PSQI scores. With regard to the genetic profiling, we found one AD patient with a known pathogenic *PSEN1* (PSEN1 p.C263F) mutation and one AD patient with the Belgian *ABCA7* founder mutation (ABCA7 p.E709Afs*85) [54, 55].

**Table 1:** Overview of participant characteristics

	**AD patients (*n* = 41)**	**Preclinical AD subjects (*n* = 8)**	**Healthy controls (*n* = 24)**	***P*-value**
Female sex	20 (49%)	4 (50%)	13 (54%)	0.942
Age at NPT	71 [67;74]	73 [71.75;74.5]	70.5 [60.75;72.25]	0.118
Years of education	14 [11;16]	15.5 [14.75;17]	14.5 [12.75;16.25]	0.266
Right handedness	38 (93%)	8 (100%)	19 (79%)	0.157
Arterial hypertension	20 (49%)	6 (75%)	5 (21%)	*0.011*^*a*^
Diabetes Mellitus	4 (10%)	0 (0%)	3 (13%)	0.736
Dyslipidemia	31 (76%)	7 (88%)	13 (54%)	0.118
Smoking	4 (10%)	1 (13%)	1 (4%)	0.571
MMSE	26 [25;28]	29 [28.75;30]	30 [29;30]	< *0.001*^*b*^
ACE-R	76 [70;80.5]	93 [87.5; 95]	93 [90;96]	< *0.001*^*c*^
GDeprS	7 [5;11]	7 [2;9.5]	3 [1;8]	0.116
Antidepressants	15 (37%)	4 (50%)	5 (21%)	0.253
Sleep medication (benzodiazepines, zolpidem, melatonin, antidepressants/psychotics)	12 (29%)	3 (38%)	2 (8%)	0.062
PSQI	4.5 [1.25;8.75]	5 [4;6.5]	4.5 [2.75;7]	0.699
TST (hours)	8.125 [7;9]	6.75 [6;7.875]	7 [6.5;8]	0.051
TST > 9 h (PSQI)	6/38 (16%)	0/7 (0%)	1/24 (5%)	0.329
TST < 6 h (PSQI)	4/38 (11%)	2/7 (29%)	4/24 (17%)	0.352
Time to sleep onset (min) (PSQI)	13.5 [5;15]	10 [7.5;13.75]	10 [5;16.25]	0.701
Whole brain volume (ml)	1402.9 [1371.6; 1451.3]	1492.9 [1461.1;1525.0]	1489.8 [1443.0; 1538.6]	< *0.001*^*d*^
Hippocampal volume (ml)	8.5 [7.7;9.2]	9.6 [8.9;10.1]	9.3 [8.6;10.5]	*0.001*^*e*^
FLAIR hyperintensity volume (ml)	5.1 [2.7;15.7]	6.0 [3.6;7.7]	2.0 [1.2;5.0]	*0.032*^*f*^
*APOE*				
No ε4	13/36 (36%)	0/6 (0%)	16/19 (84%)	< *0.001*^*g*^
One ε4	16/36 (44%)	5/6 (83%)	3/19 (16%)	*0.009*^*h*^
Two ε4	7/36 (19%)	1/6 (17%)	0/19 (0%)	0.101

Data is reported as number or median with IQR [Q1,Q3], as appropriate. *P*-values were calculated with Fisher’s exact and Kruskal–Wallis tests (with post hoc Mann–Whitney *U* tests and Bonferroni correction if statistically significant differences were found). Significance level was set at 0.05

ACE-R scores were missing in 10 AD patients, in 1 preclinical AD subject and in 3 healthy controls. GDeprS was missing in 12 AD patients, 1 preclinical AD subject and 3 healthy controls. PSQI was missing in 3 AD patients and 1 preclinical AD subject. Whole brain volume and hippocampal volume were missing in 3 AD patients, 1 preclinical AD subject and in 2 healthy controls. FLAIR hyperintensity volume is missing in 14 AD patients, in 1 preclinical AD subjects and in 6 healthy controls

*NPT* neuropsychological testing, *MMSE* Mini Mental State Examination, *ACE-R* Addenbrooke’s Cognitive Examination—Revised, *GDeprS* Geriatric Depression Scale, *PSQI* Pittsburgh Sleep Quality Index, *TST* total sleep time, *FLAIR* fluid-attenuated inversion recovery, *APOE* apolipoprotein E

Significant difference was found between following groups, after Mann–Whitney *U* test with Bonferroni correction:

^a^Healthy controls versus preclinical AD subjects: *p* = 0.03

^b^Healthy controls versus AD patients: *p* < 0.001; preclinical AD subjects versus AD patients: *p* < 0.001

^c^Healthy controls versus AD patients: *p* < 0.001; preclinical AD subjects versus AD patients: *p* = 0.003

^d^Healthy controls versus AD patients: *p* < 0.001

^e^Healthy controls versus AD patients: *p* = 0.006; preclinical AD subjects versus AD patients: *p* = 0.03

^f^Healthy controls versus AD patients: *p* = 0.033

^g^Healthy controls versus AD patients: *p* < 0.001; healthy controls versus preclinical AD subjects: *p* < 0.001

^h^Healthy controls versus preclinical AD: *p* = 0.018

### SEA

#### Prevalence of SEA

**Fig. 1:**
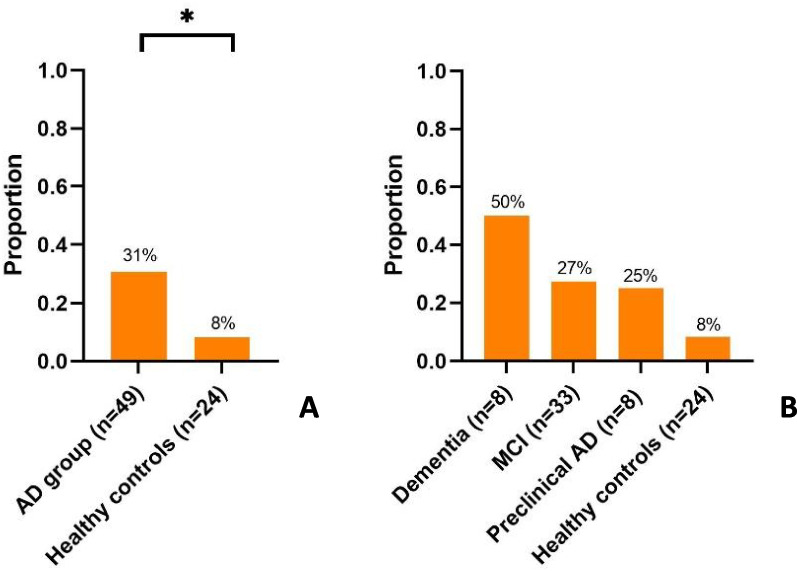
Prevalence of SEA in the AD group versus healthy controls (**A**) and in dementia due to AD patients, MCI due to AD patients, preclinical AD subjects and healthy controls separately (**B**), as detected by LTM-EEG and/or hd-EEG and/or MEG

As shown in Table 2 and Fig. 1A, SEA was detected in 31% of participants belonging to the AD group and in 8% of healthy controls, reaching statistical significance. Table 2 furthermore shows that we found a trend towards increases in detectability of SEA and spike number with all the different techniques in the AD group as compared to healthy controls, however without reaching statistical significance. As shown in Fig. 1B, we detected SEA in 4 out of 8 dementia patients (50%), 9 out of 33 MCI patients (27%), 2 out of 8 preclinical AD subjects (25%) and in 2 out of 24 healthy controls (8%) (*p* = 0.068; Fisher’s exact test). In the AD group, the median percentage of artefact containing LTM-EEG time with regard to the total LTM-EEG time was 22% [17%; 31%], in the healthy control group 21% [15%; 23%].

**Table 2:** Prevalence of SEA and IED number in the AD group and healthy controls

	**AD group**	**Healthy controls**	***P*-value**
SEA [SEA, as expressed per total amount of dementia/MCI/preclinical AD participants]	15/49 (31%) [4/9/2 **/** 8/33/8]	2/24 (8%)	*0.041*
Based upon LTM-EEG	9/48 (19%) [4/4/1** /** 8/32/8]	1/23 (4%)	0.151
Based upon hd-EEG	5/26 (19%) [0/5/0 **/** 2/19/5]	1/13 (8%)	0.643
Based upon MEG	4/16 (25%) [2/1/1 **/** 3/10/3]	0/7 (0%)	0.273
IED number			
Based upon LTM-EEG (median: 23.5 h)	5 [3;8]	4	0.726
Based upon hd-EEG (50 min)	3 [3;4]	2	0.221
Based upon MEG (50 min)	64.5 [7.75;131.5]	0	N/A

Data is reported as number or median with IQR [Q1,Q3], as appropriate. *P*-values were calculated with Fisher’s exact and Mann–Whitney *U* test. Significance level was set at 0.05. Spike number based upon MEG could not be compared between the AD group and healthy controls, as no spikes were found in the healthy control group, therefore this is not applicable (N/A)

### Best technique to detect SEA in the AD group

Table 3 shows how detectability of SEA and spike number per 50 min vary across the three different techniques. We could not find a significant difference regarding the detectability of SEA. The spike number, however, was significantly higher with MEG as compared to LTM-EEG (*p* = 0.015; Mann–Whitney *U* test with Bonferroni correction) and hd-EEG (*p* = 0.042; Mann–Whitney *U* test with Bonferroni correction).

**Table 3:** Comparison of SEA and spike number in AD as found with LTM-EEG, hd-EEG and MEG

	**LTM-EEG**	**HD-EEG**	**MEG**	***P*-value**
SEA	9/48 (19%)	5/26 (19%)	4/16 (25%)	0.879
Number of spikes per 50 min	0.19 [0.17;041]	3 [3;4]	64.5 [7.75;131.5]	*0.002*^a^

Data is reported as number or median with IQR [Q1,Q3], as appropriate. *P*-values were calculated with Fisher’s exact and Kruskal–Wallis with Mann–Whitney *U* test and Bonferroni correction if statistically significant differences were found. Significance level was set at 0.05. To calculate the number of spikes per 50 min for LTM-EEG, the calculation as described in the “Methods” Sect. (Formulas) was used

^a^MEG versus LTM-EEG: *p* = 0.015; MEG versus hd-EEG: *p* = 0.042

**Fig. 2:**
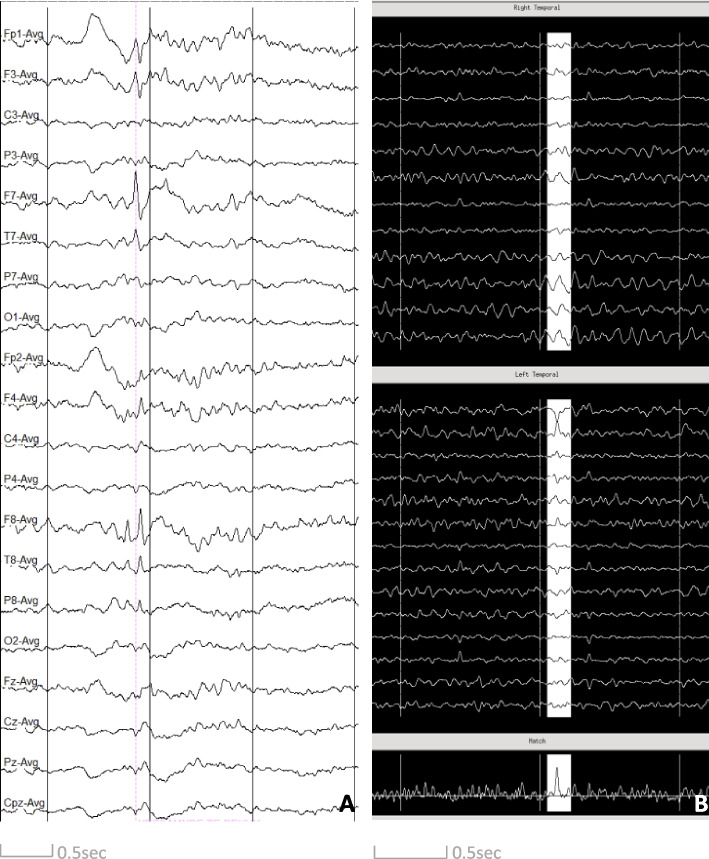
Two examples of a left frontotemporal spike on LTM-EEG (**A**) in average montage and left temporal spike on MEG (**B**)

Table 4 shows an overview of all participants with SEA, with the number of spikes found per technique and with the localisation of SEA. In most participants IEDs were seen with only one technique, whereas in 3 participants we found IEDs both with LTM-EEG and MEG. It has to be kept in mind that 2 participants in the AD group were under benzodiazepine treatment during the study (ESN4, ESN14). One of them (ESN4) took clonazepam for REM sleep behaviour disorder and one (ESN14) took lorazepam for anxiety. Both of these participants showed SEA with hd-EEG or MEG, respectively. When looking at the 10 participants belonging to the AD group in whom SEA was found and both EEG (either LTM-EEG or hd-EEG) and MEG were available, EEG was the only modality to detect SEA in 6 participants (60%), MEG was the only modality to detect SEA in 1 participant (10%) and both detected SEA in 3 participants (30%). Figure 2 shows examples of epileptic spikes found on LTM-EEG and on MEG in two different AD patients.

**Table 4:**
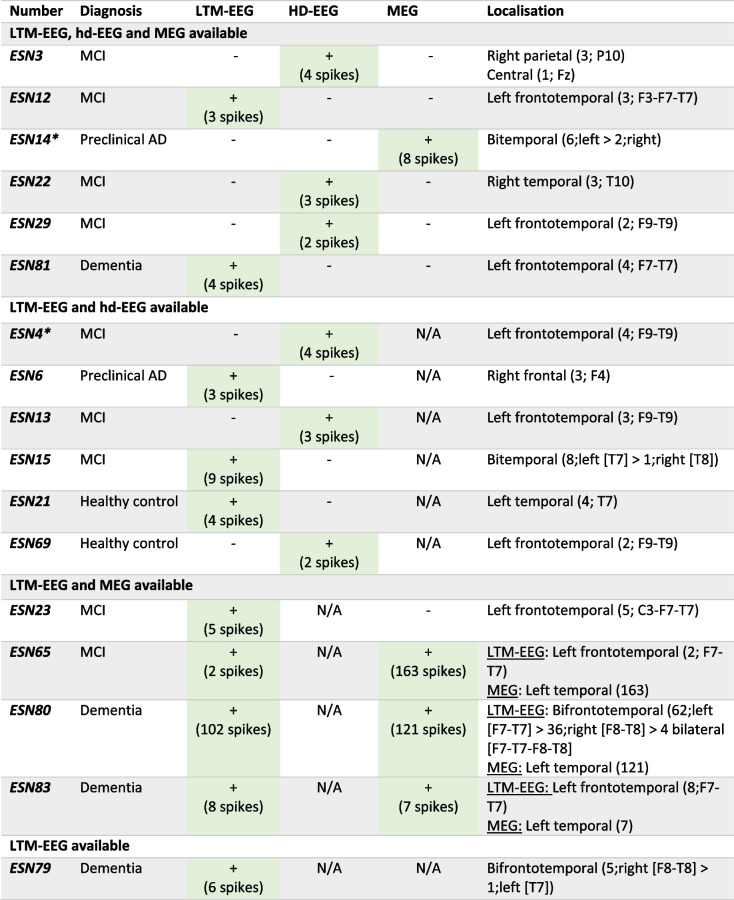
Overview of participants with SEA, with detection technique of and localisation of SEA

The localisation highlights the region where SEA was seen and for (hd- and LTM-)EEG the electrodes on which it was seen

*N/A* not applicable, indicating that the investigation did not take place in the participant

^*^Patients under benzodiazepine treatment

### Distribution of SEA: localisation and timing

**Fig. 3:**
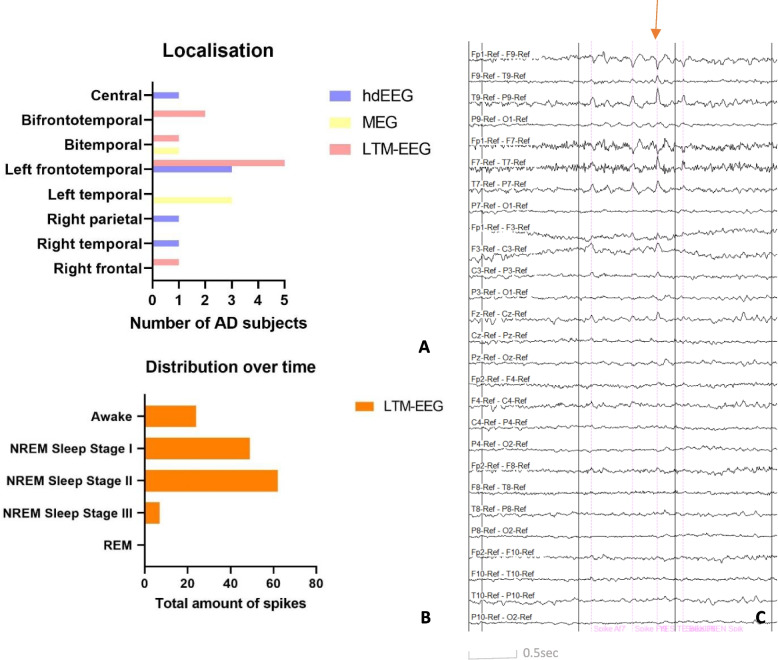
Localisation (**A**) and distribution of spikes over time (**B**) in the AD group, as well as an example of a left frontotemporal spike, maximal over the left inferior temporal chain in an AD patient in Triple Banana Montage (**C**)

Figure 3A shows the distribution of SEA over the scalp in the AD group. We found SEA most over the (fronto)temporal regions by use of all techniques and found it more over the left side than over the right side. With regard to the hd-EEG recordings, all spikes were seen most prominently over the inferior temporal chain*.* Out of the 16 spikes found in the AD group with hd-EEG, 5 would not be visible without the inferior temporal chain. Figure 3C shows an example of a left frontotemporal spike seen mostly over the left inferior temporal chain on hd-EEG. Figure 3B shows when the spikes occurred in the AD group with regard to the awake or sleep stage, further subdivided in the different sleep stages. IEDs occurred most commonly during sleep, and during sleep most in NREM sleep stage II.

### Characterisation of AD patients with SEA

For the following part of the study, we only included AD patients (MCI due to AD and dementia due to AD patients).

#### Clinical characterisation

Table 5 shows an overview of clinical characteristics in AD patients with and without SEA. We could not find any statistically significant differences regarding these characteristics between patients with and without SEA. We had one patient with a pathogenic *PSEN1* mutation and one patient with a pathogenic *ABCA7* mutation. Neither of them had SEA. There were no significant differences regarding absence, presence of one or presence of two *APOE* ε4 alleles between our two groups. Genetic profiling was missing in five patients without SEA.

**Table 5:** Clinical characterisation of AD patients with SEA and those without

	**SEA (*n* = 13)**	**No SEA (*n* = 28)**	***P*-value**
Age at NPT	68 [67;75]	71 [68;73.25]	0.448
Age at disease onset	65 [61;72]	68 [65;71.5]	0.448
Disease duration (years)	3 [3;4]	3 [2;4]	0.678
Education duration (years)	16 [12;17]	14 [10.5;15]	0.167
Female sex	7 (54%)	13 (46%)	0.744
Arterial hypertension	5 (38%)	15 (54%)	0.505
Dyslipidemia	11 (85%)	20 (71%)	0.458
Smoking	1 (8%)	3 (11%)	1.000
Diabetes mellitus	2 (15%)	2 (7%)	0.579
Anti-AD medication	10 (77%)	26 (93%)	0.304
Sleep medication	3 (23%)	9 (32%)	0.719
Antidepressants	5 (38%)	10 (36%)	1.000
TST (hours)	8.25 [7;8.87]	8.125 [6.8125;9]	0.900
Time to sleep onset (minutes)	10 [5;15]	15 [8.125;17.25]	0.347

Data is reported as median with IQR [Q1,Q3] or number as appropriate. *P*-values were calculated with Fisher’s exact and Mann–Whitney *U* test. Significance level was set at 0.05

*TST* total sleep time, TST and time to sleep onset were missing in 1 patient with and 2 patients without SEA

#### Neuropsychological characterisation

Table 6 shows an overview of neuropsychological testing scores in AD patients with and without SEA. Patients with SEA scored worse on the RBANS attention and RBANS visuospatial subset (*p* = 0.05 and *p* = 0.009, respectively) as compared to those without SEA. There were no other statistically significant differences between both groups.

**Table 6:** Neuropsychological test results of AD patients with SEA versus those without SEA

	**SEA (*n* = 13)**	**No SEA (*n* = 28)**	***P*-value**
ACE-R	76 [69;83]	75 [72;79]	0.948
ACE-R Orientation	9.5 [9;10]	9 [8;10]	0.211
ACE-R Concentration	5 [4.25;5]	5 [5;5]	0.244
ACE-R Memory	19 [16;20]	17 [16;20.75]	0.742
ACE-R Fluency	6 [3;8]	5 [3;9.5]	1.000
ACE-R Language	26 [24;26]	25.5 [22;26]	0.760
ACE-R Praxis	5 [4.25;8]	7 [6;7]	0.648
ACE-R Perception	8 [7;8]	8 [8;8]	0.257
MMSE	27 [24;28]	26 [25;27.25]	0.854
TMT-A	51 [35;72.5]	44 [30;63.5]	0.367
TMT-B	150 [109.5; > 300]	146 [117; > 300]	0.985
RBANS	80 [62.75;84]	78 [72.5;82.5]	0.868
RBANS Immediate Memory	85 [66;87]	69 [61;73]	0.101
RBANS Visuospatial	92 [84.75;108]	112 [102;116]	*0.009*
RBANS Language	87.5 [82;91.5]	89 [83.25;92]	0.811
RBANS Attention	77 [66.75;85.75]	86.5 [79;91.75]	*0.050*
RBANS Delayed Memory	64 [54;82.5]	58 [47;71.25]	0.314
GDeprS	7 [5;10]	7 [3.25;11.75]	0.635
PSQI	3 [1;8.25]	7.5 [1.75;13.25]	0.485

Data is reported as median with IQR [Q1,Q3] or number as appropriate. *P*-values were calculated with Fisher’s-exact and Mann–Whitney *U* test

Significance level was set at 0.05. Total ACE-R, ACE-R memory, ACE-R Fluency, ACE-R Language and ACE-R Perception values were missing in 4 patients with and 6 patients without SEA, ACE-R Orientation, ACE-R Concentration and ACE-R Praxis were missing in 3 patients with and 6 patients without SEA, TMT values in 2 patients with and 5 patients without SEA RBANS values in 3 patients with and 12 patients without SEA, GDeprS in 2 patients with and 10 patients without SEA and PSQI in 1 patient with and 2 patients without SEA

*MMSE* Mini Mental State Examination, *ACE-R* Addenbrooke’s Cognitive Examination—Revised, *RBANS* Repeatable Battery of the Assessment of Neuropsychological Status, *GDeprS* Geriatric Depression Scale, *PSQI* Pittsburgh Sleep Quality Index

#### Biomarker characterisation

We compared CSF biomarkers (P-tau181, T-tau, Aβ1-42 and Aβ_1-42_/Aβ_1-40_ ratio) between AD patients with SEA and those without SEA. Comparison was done within a group of patients whose CSF was analysed by use of automated ELISA at UZ Brussel (*n* = 8 with SEA, *n* = 8 without SEA), and in a group of patients whose CSF analysis was done by use of automated CLIA at UZ Brussel (*n* = 1 with SEA; *n* = 16 without SEA)*.* There were no significant differences regarding CSF biomarkers between both groups.

#### Imaging characterisation

We compared all volumes (T1-hypointensity volume, [posterior and posterior periventricular] FLAIR hyperintensity volume, whole brain volume, grey matter volume, cortical grey matter volume, white matter volume, frontal cortex volume, parietal cortex volume, temporal cortex volume, occipital cortex volume, cingulate cortex volume, anterior cingulate cortex volume, posterior cingulate cortex volume, entorhinal cortex volume, hippocampal volume, parahippocampal volume, precuneus volume, fusiform volume with left and right side volume if applicable) between AD patients with SEA and AD patients without SEA. We found higher left frontal, (left) temporal and (left and right) entorhinal cortex volumes in AD patients with SEA as compared to those without. Table 7 displays all statistically significant differences, as well as the (posterior and posterior periventricular) FLAIR hyperintensity volume and T1 hypointensity volume in both groups.

**Table 7:** Comparison of selected MRI volumes between AD patients with SEA and those without

	**SEA (*n* = 13)**	**No SEA (*n* = 25)**	***P*-value**
Frontal cortex volume left (ml)	105.0 [100.5;111.98]	97.4 [93.2;102.9]	0.038
Temporal cortex volume (ml)	132.2 [125.2;137.2]	122.5 [112.0;131.0]	0.041
Temporal cortex volume left (ml)	67.9 [62.6;69.4]	62.2 [54.2;63.8]	0.041
Enthorinal cortex volume (ml)	5.3 [4.9;5.6]	4.6 [4.0;5.1]	0.027
Enthorinal cortex volume left (ml)	2.6 [2.5;2.8]	2.4 [2.1;2.5]	0.041
Enthorinal cortex volume right (ml)	2.6 [2.3;2.8]	2.3 [2.0;2.5]	0.032
FLAIR hyperintensity volume (ml)	9.4 [4.4;14.2]	4.0 [2.6;15.7]	0.524
FLAIR hyperintensity volume, posterior region (ml)	2.9 [1.5;6.8]	2.0 [0.9;8.5]	0.381
FLAIR hyperintensity volume, posterior periventricular region (ml)	2.5 [1.4;6.5]	1.95 [0.85;8.1]	0.541
T1 hypointensity volume (ml)	3.0 [2.2;6.2]	3.2 [2.6;8.4]	0.590

FLAIR hyperintensity volume and T1 hypointensity volume were not significantly different but are shown as well. Data is reported as median with IQR [Q1,Q3]. *P*-values were calculated with Mann–Whitney *U* test

Significance level was set at 0.05

FLAIR hyperintensity volumes were missing in 5 patients with SEA and in 6 patients without SEA. All volumes were missing in 3 patients without SEA

*FLAIR* Fluid Attenuated Inversion Recovery

## Discussion

Neuronal hyperactivity and hyperexcitability are inextricable features of AD, with a potential contributing role of hallmark AD proteins Aβ and tau [6–9, 56]. Next to hallmark AD proteins, neuroinflammation, cerebrovascular, cytoskeletal and structural changes might contribute to seizure susceptibility in AD [6, 57]. Because hallmark AD proteins are present years before symptom onset [5], we wanted to evaluate the prevalence of SEA in the AD continuum, including the preclinical AD stage [5]. Using LTM-EEG, hd-EEG and MEG, SEA was detected in 31% of participants belonging to the AD group and in 8% of healthy controls, reaching statistical significance. The AD group consisted of 8 patients with dementia due to AD patients, 33 patients with MCI due to AD and 8 preclinical AD subjects according to NIA-AA 2011 research criteria using biomarkers (reflecting high probability of underlying AD pathophysiology) [5, 14, 15]. The evaluation of SEA in AD cohorts based upon research criteria using biomarkers, giving the possibility to recruit preclinical AD subjects, has to our knowledge not been done before. Comparing the different groups, we found SEA in 50% of AD dementia patients, 27% of MCI due to AD patients and 25% of preclinical AD subjects. Although differences in these proportions across these three subgroups did not reach statistical significance, these results might suggest that the presence of SEA increases with disease progression, which is consistent with previous literature that states that seizure risk increases with more severe disease [18]. However, there was no significant difference in disease duration between our AD patients with SEA and those without. This might be explained by the fact that we included mostly MCI due to AD patients (more than dementia due to AD patients), who might per se have shorter disease durations than dementia patients, making it harder to pick up statistical differences regarding disease duration between AD patients with SEA versus those without.

Our findings are in line with findings by Lam et al. who found epileptiform abnormalities in 22% of AD-NoEp participants by use of LTM-EEG. When only considering LTM-EEG, we found a prevalence of SEA in 19% of the AD group. In our study, we used spikes as a marker of SEA, whereas Lam et al. also looked at other EEG markers for epileptiform activity such as *temporal intermittent rhythmic delta activity (TIRDA)* [11]. Our findings are also in line with the findings of Vossel et al. who found SEA in 21.2% of AD patients by use of LTM-EEG [12]. On the other hand, Brunetti et al. described the absence of a significant difference regarding epileptiform abnormalities between probable AD patients, MCI due to AD patients and healthy controls. However, they only considered one to have epileptiform activity if they found at least 10 spikes during their full-night polysomnography, potentially contributing to the lower number of participants belonging to the groups with epileptiform abnormalities [58]. We detected SEA in 8% of healthy controls by use of hd-EEG and in 4% by use of LTM-EEG. This is slightly above (for hd-EEG) or completely within (for LTM-EEG) the previously reported 0–6.6% of spontaneous IEDs in healthy adults without previous seizures, although the longer duration of LTM-EEG and extended head coverage of hd-EEG in our study should be taken into account [59]. The number of spikes found in our AD group is low, with a median spike number of 5 spikes per LTM-EEG. This is in line with findings by Lam et al. who described a median frequency of 3 spikes per 24 h EEG in the AD-NoEp group [11]. Vossel et al. described a spike frequency in AD patients of 0.03 to 5.18 per hour, or alternatively 0.72 to 122 spikes per 24 h, in AD patients without seizures [12]. This is in line with our spike frequency ranging between 2 and 102 spikes per LTM-EEG in the AD group. We found spikes mostly in the (fronto)temporal regions, left more than bilateral more than right, which is in line with finding by Horvath et al. who found spikes predominantly in the temporal regions (left > bitemporal > right) by use of EEG. Lam et al. and Vossel et al. also found spikes predominantly in the left temporal region by use of EEG; however, Vossel et al. found spikes more in the right than left temporal regions by use of MEG, which is not in line with our findings [11–13]. Lam et al. described different localisation of epileptiform activity in the AD-Ep group versus the AD-NoEp group. In the AD-NoEp patients, discharges were mostly found in the left temporal lobe but also bifrontal. In the AD-Ep group, epileptic discharges were seen in both left and right temporal regions [11]. As opposed to the papers by Lam et al., Vossel et al. and Horvath et al., we began our EEG analysis pipeline with an automated spike detection method (Persyst Spike Detector P14 [Persyst, San Diego, CA, U.S.A.]). The previous Persyst software package (Persyst 13) has been proven to be non-inferior to humans when calculating the Spike Wave Index in electrical status epilepticus in sleep [60]. Another study showed that IED detection by Persyst 14 is similar to human review, when reviewing 30-min selections and 10-s epochs [44].

Hd-EEG has not been used in AD before to detect epileptiform abnormalities. We wanted to evaluate on the potential added value of the inferior temporal chain in AD because the standard 10–20 EEG system insufficiently records activity from the mesial temporal structures [30]. Using hd-EEG, we found SEA in 5 out 26 participants belonging to the AD group. All spikes recorded with hd-EEG were most visible over the inferior temporal chain, with some only being visible over this chain. Out of the 16 spikes found on hd-EEG in our AD group, 5 would not be visible without having the inferior temporal chain when reading the EEG. As there were no significant differences in the prevalence of SEA or spike number found by LTM-EEG versus 50-min hd-EEG in AD, we thought it might be worth considering only doing a short EEG with inferior temporal chain when chasing SEA in AD. However, in those AD participants in whom we found SEA and both LTM-EEG and hd-EEG were available, hd-EEG was able to detect SEA in 5 AD participants in whom LTM-EEG did not, and LTM-EEG was able to detect SEA in 4 AD participants in whom hd-EEG did not. Therefore, these techniques remain complementary in detecting SEA in AD. Adding the inferior temporal chain to the standard LTM-EEG caps/nets/montages could be a potentially interesting way forward, concluding two examinations into one, leading to a higher SEA detection yield in AD.

The prevalence of SEA was not higher with one neurophysiological technique as compared to the other. The number of spikes, however, as found by MEG were significantly higher than by use of EEG. MEG and EEG both have their own (dis)advantages but remain complementary: MEG has a higher spatial resolution than EEG (but only for superficially arising signals) and is highly sensitive for tangential sources arising in the walls of cortical fissures. On the other hand, EEG is sensitive to radial currents which is not the case for MEG [27]. It has already been shown that MEG detected interictal spikes in 19 out of 22 patients with intractable mesial temporal lobe epilepsy [61]. Carrette et al. described presence of IEDs on MEG in 26 out of 38 patients (68%) with presumed MTLE with all spikes situated in the temporal lobe [62]. Vossel et al. found a higher prevalence of SEA by use of MEG (33.3%) as compared to LTM-EEG (21.2%) in their AD patients. The spike frequency was 1 to 20 per hour on MEG, which is lower than the amount of spikes that we found using MEG (1 to 163) [12]. It should be kept in mind that MEG is less accessible than EEG [63]. The experience in MEG analysis and interpretation is relatively constrained to specialised institutions with the necessary technology and experienced personnel [64]. As it seems rather hard to pick up epileptiform activity from the mesial temporal region by use of EEG only in AD patients, Lam et al. evaluated the use of foramen ovale electrodes in two AD patients. They were able to detect silent hippocampal seizures and spikes in these AD patients without a history or EEG evidence of seizures [65]. Furthermore, machine learning techniques to detect epileptiform activity arising from the hippocampus are currently being developed [26, 66].

Previous research showed that AD patients with epileptiform activity/seizures were significantly younger and had a younger age of disease onset than AD patients without [20, 67, 68]. We could not confirm this in our study. Our group of patients with SEA, however, scored lower on the RBANS visuospatial and attention subset score than those without. This is partially in line with findings by Horvath et al. who found lower scores on the visuospatial subset of the ACE-R in AD patients with epileptiform activity [13]. However, they also found lower scores on the ACE-R memory subset [13], which we could not confirm. In another study, Horvath et al. found that AD patients with seizures performed worse in visuospatial scores than those without seizures [21]. Lower visuospatial subset scores in patients with epileptiform activity might be consistent with the fact that AD patients with generalised motor seizures have significantly more neuronal loss and brain atrophy, specifically in regions with large pyramidal cells such as the parietal cortex [69, 70]. Smaller parietal thickness with reduced volume of both precunei, as well as smaller volumes in the right parahippocampal gyrus, left angular gyrus and middle temporal gyrus in a group of AD patients with seizures as compared to the AD group without seizures has been described [21, 22]. Despite the lower scores on the RBANS visuospatial subset, our AD patients with SEA however did not have lower parietal volumes than those without. It must be underlined that our patients furthermore do not have (generalised motor) seizures, which was associated with significantly more neuronal loss in the parietal cortex [69, 70]. It has been shown that AD patients have more posterior predominant (i.e. parieto-occipital and posterior periventricular) WMH as compared to the normal aging population [71]. This is why we compared the amount of WMH in the posterior and posterior periventricular regions between AD patients with SEA versus those without to evaluate whether these WMH could potentially contribute to more severe visuospatial deficits in AD patients with SEA. Although we could not find any significant differences in WMH within these regions between our group of AD patients with SEA as compared to those without, this might be due to a lack in power, as we only had FLAIR hyperintensity volumes available for 8 AD patients with SEA and 19 without SEA. IEDs per se might also lead to transient cognitive impairment, as has been seen in epilepsy patients [72]. Transient cognitive impairment is characterised by a temporary deficit in memory encoding, attention, communication, or visuospatial abilities [73], which could therefore contribute to the lower RBANS attention and visuospatial abilities in AD patients with SEA.

Although we found trends towards a higher prevalence of SEA already in preclinical AD subjects, which might further underline the role of hallmark AD proteins in inducing SEA, we could not find any significant differences regarding the CSF biomarker data between AD patients with SEA versus those without. We might have expected lower Aβ_1-42_/Aβ_1-40_ ratio and Aβ_1-42_ and/or higher P-tau181 and T-Tau levels in our AD patients with SEA, given the role of Aβ and tau in inducing neuronal hyperactivity [6]. However, we do lack power for this analysis, with the comparison within the CLIA group only entailing one patient with SEA. Our AD patients with SEA have larger left frontal, (left) temporal and (left and right) entorhinal cortex volumes than those without, which has not been described before. Although the exact cause of these larger volumes is not clear (yet), we hypothesise that not only neurodegeneration and neuronal loss might play a role in epilepsy in/and AD. Other biological changes have been described to occur during epileptogenesis, amongst which gliosis and aberrant neurogenesis [74]. Whether gliosis is cause or consequence of epilepsy is under debate, but it has also been described as hallmark of AD [74, 75]. One of its defining features includes hypertrophy of cell bodies and processes of glial cells, potentially contributing to increased volumes as found in our study [74]. In our study, increased MRI volumes were seen in those regions where IEDs were also found, which could be cause or consequence of gliosis in these regions. Whether gliosis and aberrant neurogenesis could explain the increase in volumes that we found in our study merits further investigations. This could potentially be done by using in vivo techniques such as translocator protein 18 kDa (TSPO) PET. TSPO is a biomarker suited for assessing active gliosis [76, 77]. Alternatively, more post-mortem anatomopathological studies in AD patients with SEA would be interesting. We furthermore could not find a statistically significant difference regarding FLAIR hyperintensity volume or T1 hypointensity volume between our AD patients with and without SEA. The volumetric data suggest that the SEA in AD cannot be solely attributed to an increase in cerebrovascular pathology or to cerebral atrophy. Although *APOE* ε4 seems to be a risk factor for epilepsy [17], we could not find any significant differences regarding *APOE* ε4 carrier status between AD patients with SEA versus those without. The *PSEN1* and *ABCA7* mutation carriers did not exhibit SEA.

Our study has several strengths and limitations. One of the strengths is the fact that we gathered a well-characterised, representative study population, as AD patients had lower MMSE and ACE-R scores, and lower whole brain and hippocampal volumes on MRI than healthy controls. Furthermore, the absence of *APOE* ε4 alleles was significantly lower in our AD group compared to controls. Another strength is that, for the readout of MEG, hd-EEG and LTM-EEG, we called on experienced neurophysiologists/epileptologists who were blinded to clinical diagnosis. Limitations of our current study are the relatively low sample size with a limited number of MEGs and hd-EEGs. Another potential drawback of the study might be the fact that we only looked at spikes as a marker for SEA. Looking at other markers for epileptiform activity, e.g. TIRDA, might further increase the detectability of epileptiform activity. It furthermore has to be noted that there is high intra- and interrater variability within and between epileptologists/clinical neurophysiologists when interpreting EEG and looking at epileptic discharges [44, 78]. Grant et al. described intrarater kappa ranging from 0.33 to 0.73 and interrater kappa from 0.29 to 0.62 when interpreting EEGs [78]. Another study described a kappa between human experts in IED detections per 30 min of 0.69 [44]. Furthermore, the distribution and location of IED were not unanimously interpreted by different experienced readers in patients with childhood idiopathic epilepsy [79]. We considered the different sleep stages in which IEDs occurred, but in older participants, different electroencephalographic elements defining different sleep stages become less well differentiated, e.g. spindle number, density and duration as well as K-complex number and density are all significantly lower in the elderly compared to young adults [80], and there are deficits in slow waves in NREM sleep with increasing age [81]. Furthermore, it has been shown that in patients with amnestic MCI, there are less sleep spindles in frontal and parietal regions and there is less delta power in central and parietal regions in NREM sleep as compared to age-matched controls, which makes it even more difficult to classify sleep stages in AD [82]. Whereas we used a Bonferroni correction for post-hoc comparisons between groups, we did not use this for multiple testing. In Table 6 and 7, comparison of multiple parameters between AD patients with SEA and those without would lead to very stringent *p*-values to attain to. In order to avoid the introduction of type II statistical errors, we did not use a Bonferroni correction for multiple testing. Therefore false-positive significant differences between our groups should be taken into account [83]. Another limitation of the study is the fact that the three recording techniques (LTM-EEG, hd-EEG, MEG) were not all systematically used in each participant. This would have given us the opportunity to even better compare the SEA detection yield in AD between these different techniques. During the course of this study, we suffered setbacks with alternating technical issues with the hd-EEG (broken amplifier), MEG (water leak in the MEG facility leading to several months of closure) and LTM-EEG (broken electrodes) as well as personal patient factors (e.g. dental artefacts on MEG, making it unreadable) waiving the possibility to perform or analyse all examinations in each participant. Furthermore, the change in LTM-EEG setup by adding Fp1 and Fp2 electrodes to our caps, after performing nine LTM-EEG experiments without these electrodes is another limitation of the study.

The presence of neuronal hyperactivity and SEA might further disrupt cognitive functions in AD. As mentioned before, transient cognitive impairment due to IEDs has been described in epilepsy patients, which might also explain sudden alterations in cognitive functioning of AD patients [84]. Both focal and generalised IEDs can disrupt cognitive performance. Abnormalities of neuropsychological test profiles increase with the frequency of IEDs [72]. Neuronal hyperactivity and SEA can also lead to disease progression due to increased production of Aβ, possibly due to increased endocytosis of APP, or by stimulating the release of tau in vivo and in vitro, leading to the spread to tau pathology [23–25]. Vossel et al. showed that patients with SEA had a faster decline in global cognitive and executive functions [12]. Horvath et al. found faster cognitive decline in AD patients with SEA, represented as a higher yearly decrease in ACE and MMSE scores [13]. Another study by Vossel et al. showed that administration of levetiracetam improved performance on spatial memory and executive function tasks in patients with AD and SEA [85]. As follow-up of the cohort is ongoing, the absence of follow-up data in the present paper makes it impossible to know the impact of SEA on the clinical progression in our study. However, the influence of SEA on cognition and cognitive decline in AD merits further investigation, as well as the potential effect of antiepileptic drugs on cognitive decline in AD.

## Conclusions

We found a higher prevalence of SEA in a group of well-defined AD subjects, including patients with dementia and MCI due to AD and preclinical AD subjects, as compared to cognitively healthy controls by use of a combination of LTM-EEG, hd-EEG and/or MEG. SEA detectability tended to increase with disease stage, was most importantly found in the (fronto)temporal regions and was associated with worse RBANS visuospatial and attention scores and increased left frontal, (left) temporal and (left and right) entorhinal cortex volumes in AD patients.

In the progression of AD, several potential pathophysiological processes are at play. A measurable and treatable cause of AD associated dysfunction is a change in neuronal excitability, leading to higher seizure susceptibility. IEDs and (sub)clinical seizures are known to be leading to cognitive problems. Since epileptic activity is amendable to treatment, determining the best way to detect IEDs in AD is of paramount importance to guide effort into further treatment.

## Data Availability

The datasets used and/or analysed during the current study are available from the corresponding author on reasonable request.

## Abbreviations

Aβ: Amyloid-beta
ACE(-R): Addenbrooke Cognitive Examination (Revised)
AD: Alzheimer’s disease
ADL: Activities of daily living
AD-Ep: AD patients with epilepsy
AD-NoEp: AD patients with no history or risk factors for epilepsy
CLIA: Chemiluminescence immunoassay
CSF: Cerebrospinal fluid
CT: Computed tomography
EEG: Electro-encephalography
ELISA: Enzyme-linked immunosorbent assay
FDG: Fluorodeoxyglucose
FLAIR: Fluid attenuated inversion recovery
(f)MRI: (Functional) magnetic resonance imaging
GDeprS: Geriatric Depression Scale
hd-EEG: High-density EEG
IEDs: Interictal epileptiform discharges
IFCN: International Federation of Clinical Neurophysiology
LTM-EEG: Long-term EEG
LP: Lumbar puncture
MCI: Mild cognitive impairment
MEG: Magnetoencephalography
MMSE: Mini-Mental State Examination
NIA-AA: National Institute on Aging and the Alzheimer’s Association
NINCDS-ADRDA: National Institute of Neurological and Communicative Diseases and Stroke/Alzheimer's Disease and Related Disorders Association.
NPT: Neuropsychological testing
NREM: Non-rapid eye movement
PET: Positron emission tomography
PSQI: Pittsburgh Sleep Quality Index
RBANS: Repeatable Battery for the Assessment of Neuropsychological Status
SEA: Subclinical epileptiform activity
TIA: Transient ischemic attack
TIRDA: Temporal intermittent rhythmic delta activity
TMT-A and -B: Trail Making Test Part A and Part B
TST: Total sleep time
VAT: Visual Association Test
WMH: White matter hyperintensitie
